# Evaluation of the Usability of a Low-Cost 3D Printer in a Tissue Engineering Approach for External Ear Reconstruction

**DOI:** 10.3390/ijms222111667

**Published:** 2021-10-28

**Authors:** Constanze Kuhlmann, Jana C. Blum, Thilo L. Schenck, Riccardo E. Giunta, Paul Severin Wiggenhauser

**Affiliations:** 1 Department of Hand, Plastic and Aesthetic Surgery, LMU Klinikum, University Hospital, LMU Munich, Ziemsenstrasse 5, 80336 Munich, Germany; jana.blum@med.uni-muenchen.de (J.C.B.); Thilo.schenck@med.uni-muenchen.de (T.L.S.); riccardo.giunta@med.uni-muenchen.de (R.E.G.); 2 Department of Breast, Plastic and Aesthetic Surgery, Frauenklinik Dr. Geisenhofer, Hirschauer Str. 6, 80538 Munich, Germany

**Keywords:** ear reconstruction, cartilage tissue engineering, 3D printing, Ultimaker, PCL, polycaprolactone, scaffold manufacturing, bioengineering, CAD/CAM, cost effectiveness

## Abstract

The use of alloplastic materials instead of autologous cartilage grafts offers a new perspective in craniofacial reconstructive surgery. Particularly for regenerative approaches, customized implants enable the surgeon to restore the cartilaginous framework of the ear without donor site morbidity. However, high development and production costs of commercially available implants impede clinical translation. For this reason, the usability of a low-cost 3D printer (Ultimaker 2+) as an inhouse-production tool for cheap surgical implants was investigated. The open software architecture of the 3D printer was modified in order to enable printing of biocompatible and biologically degradable polycaprolactone (PCL). Firstly, the printing accuracy and limitations of a PCL implant were compared to reference materials acrylonitrile butadiene styrene (ABS) and polylactic acid (PLA). Then the self-made PCL-scaffold was seeded with adipose-tissue derived stem cells (ASCs), and biocompatibility was compared to a commercially available PCL-scaffold using a cell viability staining (FDA/PI) and a dsDNA quantification assay (PicoGreen). Secondly, porous and solid patient-customized ear constructs were manufactured from mirrored CT-imagining data using a computer-assisted design (CAD) and computer-assisted manufacturing (CAM) approach to evaluate printing accuracy and reproducibility. The results show that printing of a porous PCL scaffolds was possible, with an accuracy equivalent to the reference materials at an edge length of 10 mm and a pore size of 0.67 mm. Cell viability, adhesion, and proliferation of the ASCs were equivalent on self-made and the commercially available PCL-scaffolds. Patient-customized ear constructs could be produced well in solid form and with limited accuracy in porous form from all three thermoplastic materials. Printing dimensions and quality of the modified low-cost 3D printer are sufficient for selected tissue engineering applications, and the manufacturing of personalized ear models for surgical simulation at manufacturing costs of EUR 0.04 per cell culture scaffold and EUR 0.90 (0.56) per solid (porous) ear construct made from PCL. Therefore, in-house production of PCL-based tissue engineering scaffolds and surgical implants should be further investigated to facilitate the use of new materials and 3D printing in daily clinical routine.

## 1. Introduction

The reconstruction of the auricle due to congenital deformities (e.g., microtia) or extended defects that are related to trauma or tumors is one of the most challenging procedures in facial reconstructive surgery [1]. In this procedure, costal cartilage is used to create a three-dimensional (3D) auricular framework that is covered with a thin, well-vascularized fasciocutaneous flap to ensure the nutritional support of the autograft, and to provide soft-tissue coverage [2,3,4]. Unfortunately, the use of autologous cartilage is limited, due to shortage of cartilage donor sites and concomitant donor site morbidity [5,6,7]. Furthermore, the anatomical shape and definition of the cartilaginous framework and the resulting cosmetic and functional outcome of the reconstruction are highly dependent on the surgeon’s expertise [8,9,10].

**Figure 1:**
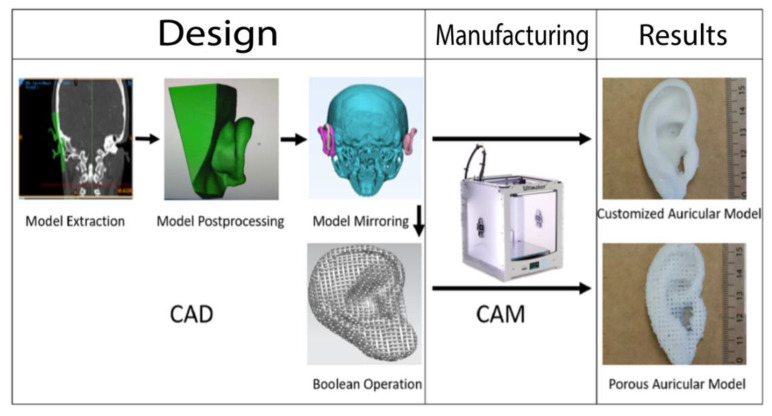
Summary of the workflow of the design and manufacturing process of the patient-customized solid and porous auricular constructs. During input phase, the unaffected ear (displayed in green) of a female patient was extracted and edited (postprocessing). The final model was mirrored with AutoCAD on a skull model, and a Boolean operation was used to design pores into the auricular construct before processing the final output with the Ultimaker 2+. The process was repeated for all three filament materials.

Hence, alloplastic reconstruction has gained momentum in reconstructive surgery during recent decades. The use of an alloplastic material represents a promising alternative to autografts that eliminates donor site morbidity and could overcome the challenge of cartilage shortage in facial reconstructive surgery [6,11]. Additionally, many alloplastic materials, such as artificial polymer compounds, are accessible for 3D printing (computer-assisted manufacturing (CAM)), which allows the fabrication of patient-customized implant geometries through computer-assisted design (CAD) approaches that describe the virtual planning from imaging data (e.g., 3D-scanning, CT, MRI) [6,12,13]. During CAD-CAM-assisted auricular reconstruction, the unaffected side can be mirrored and serve as a template for implant-processing of the affected ear (Figure 1).

Although 3D printing of an ear framework could open auricular reconstruction to less experienced surgeons, since it reduces the complexity of the procedure, high costs of commercial providers and high-end printers diminish the clinical application of 3D printed implants. In particular, in a diagnosis-based revenue system like the German Diagnosis Related Groups (G-DRG) system, the revenue of a surgical procedure is mainly based on the related diagnosis, and merely on the performed procedures. Hence, it somehow neglects the resource expenditure of experimental approaches, making them financially unattractive for clinical translation (Table 1). In-house manufacturing using a low-cost 3D printer and a low-cost filament material that fulfills the requirements for medical implications could overcome the monetary barrier, and offer an easily accessible alternative.

The Ultimaker 2+ (Ultimaker, Utrecht, The Netherlands, 2309 €) is a fused deposition modeling (FDM) technology-based standalone 3D printer that was released under a Creative Commons BY-NC (noncommercial) license. The term FDM describes the fabrication of a 3D-model using a layer-by-layer deposition of a thermoplastic material. This technique allows for the manufacture of highly reproducible scaffolds with an interconnected pore-network to allow cell migration [14,15]. The Ultimaker 2+ supports various filament materials, including acrylonitrile butadiene styrene (ABS) and polylactic acid (PLA), which are the most widely used in hobbyist desktop 3D printing [16].

Polycaprolactone (PCL) (e.g., 3D4MAKERS, Haarlem, The Netherlands, 105 €/750 g), however, is a biocompatible and biodegradable synthetic polymer that is approved by the Food and Drug Administration (FDA) for both load bearing and non-load bearing tissue engineering applications [17,18,19,20]. Moreover, the material is suitable for 3D printing, and has biomimetic properties of cartilage that have shown high regenerative potential in vitro and in vivo if seeded with chondrocytes or stem cells [19,20,21,22]. Those features and the low price make PCL an extremely interesting filament material for additive manufacturing- assisted tissue engineering approaches regarding facial cartilage. 

The purpose of this study was to evaluate the usability of a low-cost in-house 3D printer for external ear cartilage tissue engineering approaches. Since the Ultimaker 2+ and PCL are not compatible in default settings, this study intended to modify the open-architecture of the 3D printer to achieve a general printability of PCL, and manufacture an eligible cell culture scaffold from PCL for further in vitro testing. Subsequently, a CAD-CAM workflow should be implemented to fabricate patient-customized solid and porous auricular constructs from mirrored CT-imaging data to assess their potential for different experimental and clinical applications (Figure 1). ABS and PLA are considered to serve as reference materials for the evaluation of the micro- and macro-dimensional 3D printing accuracy of the self-made PCL-prototypes.

## 2. Results

### 2.1. Scaffold Modifications during the CAD-CAM Process

**Figure 2:**
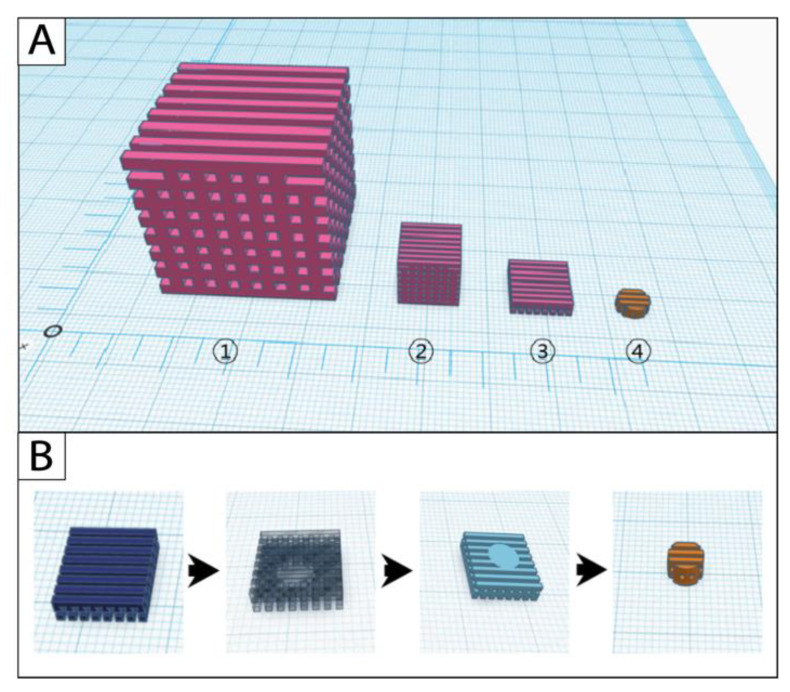
Simulations of the (**A**) scaffold modification and (**B**) postprocessing steps in the software Tinkercad. 1: original scaffold, 2: scaling scaffold, 3: model scaffold, 4: cell culture scaffold.

Design and manufacturing of the original cube scaffolds were successful with all three filament materials. The scaffold modifications started from the cube-sized original scaffold (edge length (length × width × height): 30 mm × 30 mm × 30 mm; pore size: 2 mm × 2 mm × 2 mm); the scaling scaffold (10 mm × 10 mm × 10 mm; 0.67 mm × 0.67 mm × 0.67 mm) was further processed with the slicing approach to the model scaffold (10 mm × 10 mm × 2.67 mm; 0.67 mm × 0.67 mm × 0.67 mm) (Figure 2A). Cylindric punch biopsies were taken from the model scaffold during postprocessing to receive the final cell culture scaffold (Figure 2B). 

### 2.2. Limit Test

**Figure 3:**
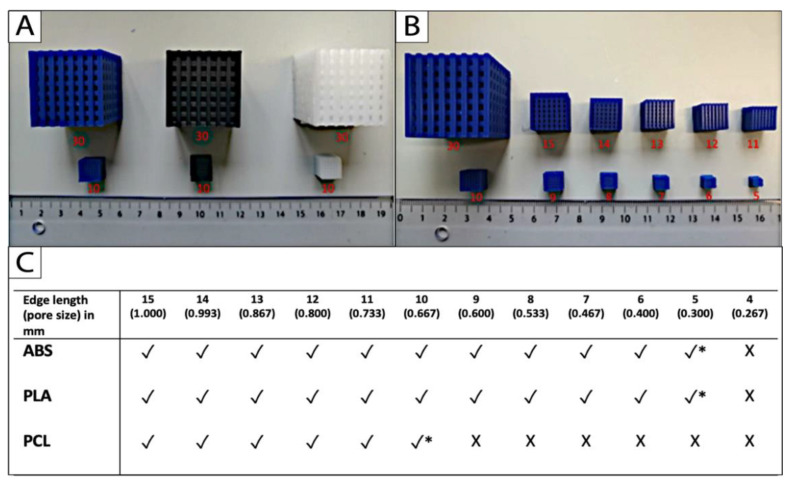
The printing limit (*) of (**A**) the filament materials ABS (blue), PLA (black), and PCL (white) was achieved (**B**,**C**) by uniform down scaling of the original scaffold, and defined as the smallest cube scaffold that could be fabricated appropriately without compromises in the micro- and macro-dimensional accuracy (*n* = 5; * printing limit; √ fabrication possible; X fabrication impossible).

Uniform scaling was applied to approach the limits of printing with the three different filament materials and the Ultimaker 2+. The limit was identified as the smallest dimension that the 3D printer could fabricate appropriately without compromising the micro- and macrostructures of the cube-scaffolds. The printing limit of PCL was achieved at a scaffold edge length of 10 mm, and a corresponding pore size of 2/3 (0.67) mm. The limit for the reference materials PLA and ABS was reached at 5 mm edge length and 1/3 (0.33) mm pore size, respectively (Figure 3B,C).

### 2.3. Micro- and Macrodimensions of the Porous Scaffolds

The accuracy and consistency of the additive manufacturing process of PCL was evaluated by measuring the micro- and macrostructures of nine scaffolds and comparing them to the results of the reference materials ABS and PLA. The approached limit of PCL (see above) was used as standard dimensions (edge length 10 mm, pore size, and width of rectangular unit (WRU) 0.67 mm), and the first 11 of 15 layers were removed with a slicing approach to receive the final model with an anticipated height of 2.67 mm (Figure 2A). The results are summarized in Table 2. No significant difference could be found in the micro- and macro-dimensions of the PCL scaffolds, compared to the reference materials (ABS and PLA) after CAD/CAM assisted manufacturing with the Ultimaker 2+.

### 2.4. Biocompatibility of the Self-Made in Comparison to the Commercially Available Scaffold 

**Figure 4:**
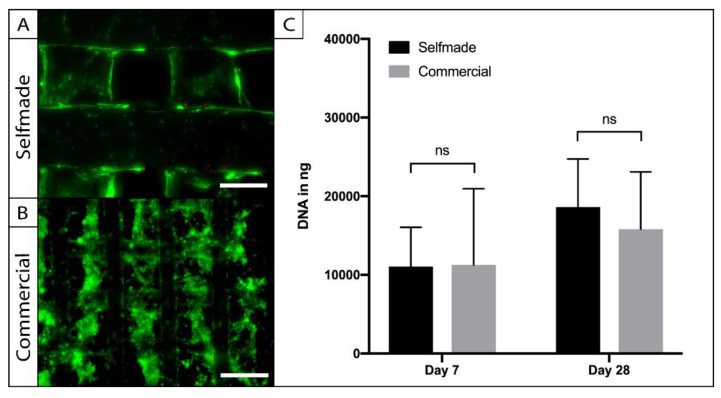
Testing for biocompatibility. ASCs were seeded on the self-made (**A**) and the commercial (**B**) scaffolds and were visualized by Live–Dead assay. Vital cells were stained in green (FDA) and dead cells are displayed in red (PI). Cell viability and adherence was comparable on the different PCL-scaffolds (A, bar = 200 µm; B = 300 µm). Absolute cell number and proliferation were evaluated with a PicoGreen assay (**C**). No statistic signifances of ASC-DNA content could be found between the compared scaffolds (*n* = 5; ns = not significant; mean ± SD).

To access the biocompatibility of the self-made PCL scaffolds, cylindric punch biopsies were cultured with adipose tissue-derived stem cells (ASCs), and compared to a commercially available ASC-seeded-PCL-scaffold in terms of cell viability, adherence, and proliferation (Figure 4). Live-Dead imaging was performed after a week in the 3D culture and revealed vital and adherent cells on the self-made and commercially available product. The cells were homogenously distributed within the pores of the scaffolds, and the pore size was large enough to allow sufficient cell migration (Figure 4A). Cell number was indirectly calculated through the amount of dsDNA within the PCL-scaffolds at the harvesting time points (Figure 4B). No significant difference could be observed between the amount of dsDNA on the compared scaffolds after 7 (*p* = 0.99) and 28 days (*p* = 0.87) of cell culture. Statistical analysis revealed a significant (*p* = 0.002) increase of cell number over time in the 3D culture, indicating cell proliferation on both PCL-scaffolds. 

### 2.5. Porous Auricular Constructs

**Figure 5:**
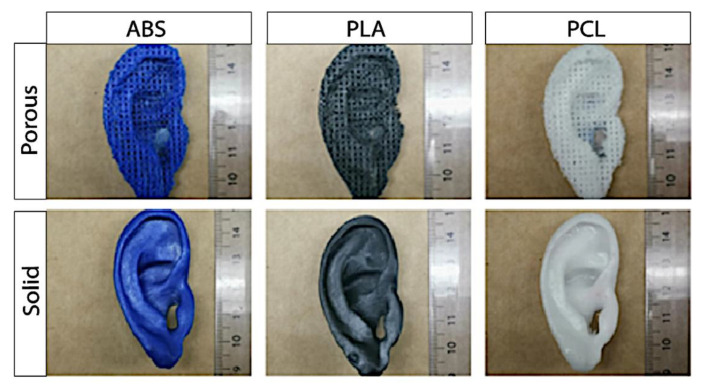
Exemplary pictures of the self-made porous (**top row**) and solid auricular (**bottom row**) constructs.

Optic assessment of the 3D printed porous constructs revealed a steady auricular implant with homogenously distributed pores (Figure 5). Palpation however, revealed sharp edges, and deficiency in the accuracy of smaller anatomical structure. In addition to the five macro-dimensions (physiognomic ear length (PEL), physiognomic ear width (PEW), morphological ear length (MEL), morphological ear width (MEW), and ear height) the micro-dimensions (pore size and WRU) were evaluated to control the precision and reliability of the Boolean operation in porous auricular constructs. Results are shown in Table 3 and Figure 5 (top row). No significant differences between ABS, PLA, and PCL could be found in statistical analysis for all measured dimensions of the 3D printed porous ear-implants.

### 2.6. Solid Auricular Constructs

The CAD/CAM process of a patient-customized ear was regarded as successful, as it was possible to create a stable and solid auricular construct from mirrored imaging data of the unaffected ear. The accuracy of five macro-dimensions of the self- and in-house-manufactured solid auricular constructs were measured and compared among the three types of filament materials. Palpation revealed a steady construct with smooth edges. The results of the statistics are shown in Table 4 and Figure 5 (bottom row). No significant difference between the accuracy of the dimensions of the final solid auricular constructs could be observed between the materials after 3D printing with the Ultimaker 2+. The printing precision of solid-PCL-constructs was comparable to solid-constructs that were manufactured from the reference materials ABS and PLA.

## 3. Discussion

The creation of the auricular cartilaginous framework remains one of the biggest challenges in facial reconstructive surgery [7,9]. To overcome this challenge, a variety of cartilage replacement materials have been developed and tested in experimental and clinical trials during recent decades [19,23]. However, most modern approaches either lack patient customization or are unattractive from an economical point of view. Particularly in additive manufacturing, expensive prices of industrial high-resolution 3D printers and prolonged delivery times of commercial implant providers stand in the way of clinical translation. Cost efficient manufacturing using a low-price 3D printer could offer a solution for the financial disproportion between the costs of implants and final surgical revenue. However, this presupposes that the quality of the self-made scaffold or implant, respectively, does not suffer as a result from low-cost production. This study addresses the chances and limitations of in-house manufacturing of scaffolds and personalized implants for facial cartilage engineering with a low-price 3D printer.

Firstly, we generated a CAD-CAM workflow to create a porous scaffold from scratch and to evaluate the potential use of the Ultimaker 3D printer for further in vitro cartilage tissue engineering. The starting point was a simple porous cube in the CAD-software that was exported to Cura software for preparation of 3D printing. Both reference materials (ABS and PLA) are compatible filaments for the Ultimaker 2+ that are cheap, easy to print, and widely favored for amateur and professional 3D printing [24]. However, for engineering of hard-tissues such as cartilage, due to its biocompatibility, slow biodegradability, and processability, PCL is the most promising material choice, but the material is not compatible with the 3D printer in default settings [25]. Through modifications in the open-source software Cura and adjustments of the printing temperature, we were able to successfully print the porous cube scaffold from PCL with the Ultimaker 2+. The scaffold dimensions were scaled down to identify the smallest size that was printable without compromising the micro- and macro-structure. Uniform scaling is a common tool in CAD that changes the overall size of an object without changing the ratio of proportions. For example, it is used to match standardized 3D printed hand- and finger prostheses to the individual residual limb volume of patients [26,27]. In our study we used the tool to test the limitations of printing and to identify the smallest printable scaffold dimensions with the in-house 3D printer. The limit test revealed an edge length of 10 mm and 5 mm, and a pore size of 0.67 mm and for PCL, and 0.33 mm for the reference materials, respectively. The presented data could be regarded as benchmark for further 3D printing approaches of porous cell culture scaffolds with the Ultimaker 2+.

Subsequently, the self-made PCL-scaffold was compared to a commercially available PCL-scaffold, which was successfully used for chondrogenic and osteogenic tissue engineering in an earlier study [28]. In vitro cultivation with human ASCs revealed comparable cell viability, distribution, and proliferation capacity on both priorly sterilized PCL-scaffolds. This makes the self-made scaffold an interesting choice for further tissue engineering approaches. Earlier studies identified a scaffold pore size within the range of native tissue (15–50 µm) as an ideal environment to maintain the differentiation of chondrocytes in cartilage tissue engineering [29,30,31]. For mesenchymal stem cells, however, Kemppainan et al. showed that increased scaffold porosity and permeability of a PCL-scaffold is more favorable for chondrogenic differentiation under the influence of induction medium [32]. This is most likely to be attributed to the more sufficient diffusion of nutrients and differentiation factors in an environment with a greater pore size and permeability [33]. The dependence on the contents of the medium apparently seems to have a greater influence on chondrogenesis of mesenchymal stem cells, such as ASCs, than cultivation in a low-permeable native-like cartilage tissue [32,34,35]. Keeping this in mind, despite being significantly larger in comparison to some other studies, the minimal pore-size of our self-made PCL scaffold (limit 0.67 mm pore size) is an acceptable choice for further experiments in cartilage tissue engineering with mesenchymal stem cells as cell source.

From an economical point of view, the production costs of the self-made PCL-scaffold, with an average weight of 0.31 g before the slicing approach rank, at EUR 0.043 cents. This makes the self-made PCL scaffold approximately 120-times less expensive than the commercially available alternative. Despite being limited in printing resolution in comparison to modern high-end 3D printers that use selective laser sintering (SLS) technology, we were able to show that the low-price 3D printer is able to produce eligible, reproducible, and biocompatible scaffolds that are highly suitable for selected in vitro tissue engineering experiments [36]. Other advantages of the self-made product are the easy access for in-house production, and the possibility to simply modify the design and structures of the scaffold to adopt to new experimental ideas and settings. For example, through adjustments in the open-source CAD-software, pore-size gradients could be designed into the scaffold to mimic zone-dependent extracellular matrix organization and manipulate the biochemical, biophysical, and biomechanical properties as was suggested for FDM-based 3D printing approaches in previous studies [33,37,38]. The design opportunities of cell culture scaffolds that come with the open-source nature of the CAD software are almost limitless within the range of resolution of the adapted additive manufacturing process with the thermoplastic materials.

For the second part of this study, we generated a workflow to manufacture low-price patient-customized anatomical ear constructs from mirrored DICOM CT-imaging data of a patient’s unaffected ear using the desktop 3D printer. Mirror image reconstruction for unilateral microtia is a feasible and reliable approach that can be conducted with a variety of imaging procedures, and improve the cosmetic and functional outcome in clinical practice [39,40,41]. We generated solid and porous auricular constructs to evaluate the potential use of the Ultimaker 2+ for manufacturing prototypes for different clinical applications, including implantation, customized tissue engineering, and surgical simulation. Besides pore size and distribution, the reproducibility and accuracy of the macro-dimensions of the constructs were evaluated by measurements of distances between predefined anatomical landmarks, whose selection was based on previous studies [42,43]. Our results show the feasibility and reproducibility of the presented CAD-CAM workflow, and revealed no significant differences in the accuracy of the dimensions between PCL and the reference materials, showing that the adjustments that were necessary to print PCL had no negative impact on the printing quality.

Recent advances in 3D printing and imaging technology make customized additive manufacturing of the cartilaginous framework of the ear a realistic goal in regenerative medicine. Kim et al. manufactured personalized implants from porous polyurethane (PU), that revealed comparable biomechanical properties to native auricular cartilage, and superior cell proliferation capacity in comparison to porous polyethylene (PPE)-based MEDPOR implants [44]. Zhou et al. were able to design and manufacture external ear scaffolds with a 3D printer that costs a manufacturer’s suggested retail price of USD $49,900, and successfully tested it in five humans [41]. The authors used CAD-CAM generated negative molds to manufacture the auricular construct that consisted of a 3D printed PCL-inner core that was wrapped in polyglycolic acid (PGA) and coated with PLA. The fabricated constructs were seeded with human auricular chondrocytes ex vivo, and revealed promising functional and cosmetic results during the follow-up period of 2.5 years [41]. In contrast to Zhou et al. we chose a rather simple and classic approach using a single material and layer-by-layer FDM for en-bloc manufacture of the porous auricular construct. The initial idea was that the porous auricular constructs could be potentially implanted as biodegradable, anatomically shaped, and patient-customized cell-seeded or unseeded scaffolds. Cell-seeded implants with patient-derived cells could reduce the risk of potential rejection of the neo-tissue, and the customized shape reflecting the anatomy of the patient’s auricle could induce cells to form tissue in the desired geometrical arrangements [40,45,46]. Furthermore, the biomimetic viscoelastic properties of PCL and the ability to integrate into native tissue without causing an inflammatory reaction represent the greatest advantages in comparison to nonabsorbable alloplastic materials, such as silicone and PPE [47,48,49,50]. The low production costs of the porous auricular PCL-construct with an average weight of 4 g amount to EUR 0.56 cents make this approach a financially attractive choice for further investigation. However, the examination of the self-made porous auricular construct revealed rather sharp edges and deficits when displaying smaller structures. This could lead to similar complications, as observed after implantation of MEDPOR implants including cosmetic deformity, subcutaneous palpation, skin protrusion, and wound healing disorders after soft-tissue coverage with a fasciocutaneous flap [51]. We conclude that the anatomy of the external ear is too filigree for porous printing approaches for in situ tissue engineering with our low-price 3D printer. Certainly, the minimal resolution of the printer could be eligible for 3D-guided tissue engineering approaches of coarser structures of the body, such as cranial or extremity bones, which should be further investigated in following studies. Additionally, the open-source software and adjustable hardware of the Ultimaker 2+ could be potentially adapted to print material blends, and further optimize the printing process of biomimetic and anatomical structures.

Moreover, complex regulatory aspects have to be considered during self-design and -production of medical scaffolds, implants, or devices. In many countries, the certification of implants is strictly controlled by national law (e.g., German law for medicinal products) that requires proof of safety and efficacy by manufacturers. This leads to high research and developmentary expenses to receive and maintain a certification for the use of a self-made implant, and impedes the step from in vitro to in situ tissue engineering and medical implantation [6]. Although legal regulations in many countries offer exceptions for therapeutical trials (e.g., in Germany “Individuelle Heilversuche”), those exceptions apply only to selected and isolated cases. In this event, the surgeon/manufacturer bears the risk for the treatment and potential claims for damages.

Within recent years, the field of surgical 3D printing has expanded its aim from creating implants and scaffolds to manufacturing 3D tools that could improve the technique and experience of surgeons by simulating the anatomy and pathology of the patient. 3D-printed surgical simulations are used during preoperative planning, for intraoperative guidance and surgical education [52,53,54]. For external ear reconstruction, different surgical simulation attempts from costal cartilage simulators to reference ears have been published using a variety of materials [10,55,56,57,58]. 3D models of reference ears aim to close the gap between the interpretation of virtual imaging data and the realistic 3D anatomy of a structure. The in-house workflow was able to generate a stable and reproducible solid auricular construct from all three filament materials. The evaluation of the macro-dimensions of the 3D-printed solid ear revealed a high degree of printing accuracy, with measurement differences below 1 mm, which is considered to be clinically undetectable [59,60]. Therefore, the solid auricular construct seems to be a promising model to simulate the ear position, and serve as a precise reference target template during construction of the autologous or alloplastic framework in auricular reconstruction surgery. The price for a comparable commercially manufactured implant is approximately USD $3000 [61]. Thus, the low-price unit prices of EUR 0.90 for PCL, EUR 0.22 for ABS and EUR 0.25 for PLA make this approach very attractive for clinical translation from an economical standpoint.

Overall, manufacturing of self-designed and anatomical PCL scaffolds and implants is possible with a modified low-cost 3D printer. Printing quality of the self-made cell culture scaffolds is acceptable for selected tissue engineering applications, and the biological activity is comparable to scaffolds from commercial providers. The desktop printer is able to produce precise patient-customized auricular models from different thermoplastic materials, with only minor deficiencies when displaying smaller structures and edges in porous implants. Manufacturing of solid auricular constructs provides sufficient anatomical accuracy for surgical simulation at a fraction of the cost from commercial providers, and could be used to simplify the complexity of auricular reconstruction. Besides the versatile clinical and experimental applications, decreasing prices of desktop 3D printers and increasing user-friendliness of CAD-software make in-house additive manufacturing a promising and valuable addition for healthcare facilities and tissue engineering laboratories. For this reason, in-house 3D printing should be pursued in the context of clinical studies in order to promote the translation from “bench-to-bedside”.

## 4. Materials and Methods

### 4.1. Computer-Assisted Design (CAD) of the Scaffold

A porous cube with an edge length of 30 mm, with eight rectangular units (pore size 2 × 2 mm) in each layer, was selected as the original shape for the scaffold. The free web-based software Tinkercad v2.0 (Autodesk, Inc., Mill Valley, CA, USA) was used to build the macrostructure from scratch. Delicate substructures (size, shape, and arrangements of pores) were added with the software AutoCAD 2018 (Autodesk, Inc., Mill Valley, CA, USA). The general structure and the distribution of the inner connections of the designed standard scaffold were evaluated with Microsoft 3D Builder (Version 1703; Microsoft, Redmond, WA, USA), and target measurements were obtained with the built-in measurement system of Microsoft 3D Viewer (Version 1703; Microsoft, Redmond, WA, USA). Data was exported in stereo-lithography file format (.STL) and imported into the open-source software Cura (Version 3.6.0, Ultimaker, Utrecht, The Netherlands) to adjust the final settings for the printing process. The final data was processed to G-code (RS-274) and transferred to the 3D printer on a micro-SD card.

### 4.2. Computer-Assisted Manufacturing (CAM) of the Scaffold

The FDM-technology based Ultimaker 2+, with extrusion upgrade kit (Ultimaker 2+, Ultimaker, Utrecht, The Netherlands), was used as the hardware unit for additive manufacturing of the final scaffold model in a “layer-by-layer” technique. 10 µm was selected as the reference layer-thickness, and 45 mm/s as the speed of the workflow. 80% was selected as the infill density of the construct, and the 0.25 mm nozzle was chosen for manufacturing. Printing temperatures were adjusted to the fabricated thermoplastics according to Table 5.

### 4.3. Uniform Scaling and Limit Test

The lowest limit of the printing process of PCL and the two reference materials (ABS and PLA) was investigated by uniform scaling of the original scaffold. Therefore, the parameters of the original scaffold were scaled down in the AutoCAD software, without changing the ratio of dimensions of the macro- and micro-structure. To approach the print limit of the Ultimaker 2+ and the different filament materials, a 15 mm cubic scaffold was scaled down systematically for 1 mm, and the limit was achieved if the 3D printer could not fabricate the scaffold properly, or if the scaffold structure collapsed after printing. The dimension of the pore size was calculated from the edge length based on the constant ratio between those two units (pore size (mm) = edge length (mm) × 1/15).

### 4.4. Evaluation of The Macro- and Microdimensions of the Cube Scaffolds

The 3D-printed test scaffolds consisted of 15 cross-sectional layers. The first 11 layers were removed using a slicing approach, leading to the final model scaffold. The macro-dimensions (edge length and height) of the manufactured scaffolds were evaluated with an electronic vernier caliper (150 mm, Wiha, Schonach, Germany). The micro-dimensions (pore size and WRU) were visualized and measured with the Axio Observer Light Microscope (Carl Zeiss, Jena, Germany) and the affiliated software, ZEN (Carl Zeiss, Jena, Germany). Nine scaffolds per filament material were produced, and each dimension was measured three times.

### 4.5. In Vitro Cultivation with Human Adipose-Tissue Derived Stem Cells (ASCs)

Human lipoaspirates were obtained from three female patients undergoing elective liposuction at the department of plastic surgery at the university hospital of the Ludwig-Maximilian University (LMU). The multipotency of ASCs was successfully demonstrated by differentiating a proportion of cells into adipogenic, chondrogenic, and osteogenic lineage, as previously published [23,62]. The in vitro experiments were performed in triplicates. All lipoaspirates were obtained through water-jet-assisted liposuction with the Body-Jet Evo system (human med AG, Schwerin, Germany). Cell isolation and culture was performed as described earlier by the authors, with a 275 U/mg collagenase type II solution (Worthington Biochemical Corporation, Lakewood, NJ, USA) [23]. ASCs were cultured in T-175 flasks (Thermo Fisher Scientific, Waltham, MA, USA) in a cell culture medium (Dulbecco’s modified eagle medium high-glucose (Thermo Fisher Scientific, Waltham, MA, USA)) supplemented with 10% fetal bovine serum (Sigma-Aldrich, St. Louis, MO, USA), 100 U/mL penicillin, and 100 µg/mL streptomycin (both from Life Technologies, Carlsbad, CA, USA)) in a humidified atmosphere (37 °C, 5% CO_2_,and 21% O_2_). The medium was changed twice a week. After reaching 80% confluency, ASCs were frozen down in passage 1 and stored in liquid nitrogen until usage for experiments. A central punch biopsy was taken from the self-made PCL-scaffolds postprocessing in order to obtain a disk-like model for cell culture. The self-made scaffolds were treated with sodium hydroxide (Merck Millipore, MA, USA) after manufacturing to improve hydrophily. Further, the scaffolds were incubated in 70% ethanol (Carl Roth, Karlsruhe, Germany). After 30 min, scaffolds were transferred into a fresh well-plate, and 1 mL of 5 M sodium hydroxide was pipetted directly onto the surface. The well-plate was moved into the incubator for 5 h, where linear shaking was performed at 37 °C. Commercially available disk-like cell culture PCL-scaffolds with square-shaped pores (BellaSeno GmBh, Leipzig, Germany) were used as the reference group. The reference-scaffolds had a height of 1 mm and a diameter of 5 mm, with a square based pore geometry with a pore size of 300 µm. Directly prior to seeding, both scaffolds were sterilized in 70% ethanol under UV-light for 2 h, and 2.5 × 10^6^ cells concentrated in cell culture medium were pipetted directly on the scaffold-surface. All scaffolds were plated in a 24-well plate (Corning, NY, USA), and were cultured under the same conditions as freshly isolated cells.

### 4.6. Testing for Biocompatibility

Cell viability was visualized with Live-Dead staining solution (containing 8 µg/mL fluorescein diacetate and 20 µg/mL propidium iodide-both Sigma Aldrich, St. Louis, MO, USA) after 7 days of 3D culture, as previously described [23]. Furthermore, the amount of dsDNA within the scaffolds was quantified with the Invitrogen™ Quant-iT™ PicoGreen™ dsDNA Assay Kit (Thermo Fisher Scientific, Waltham, MA, USA) after 7 and 28 days. Scaffolds were harvested and prepared as described by Wiggenhauser et al. [19]. For the fluorometric measurement of the samples, Tris-EDTA-Buffer was added in a ratio of 1:3, and 100 µL of the solution were diluted 1:1 with Quant IT PicoGreen dsDNA reagent. Fluorescence was measured in a black bottom 96-well plate (Corning, NY, USA) at 504 nm extinction and 550 nm emission wavelength with a plate reader (Tecan SAFIRE II, Tecan Group, Maennedorf, Switzerland). The volume of DNA was calculated in ng/mL against a dsDNA standard curve that was prepared of a serially diluted Lambda DNA standard.

### 4.7. Computer-Assisted Design (CAD) of Customized Porous and Solid Auricular Constructs

Anonymized data from CT-imaging of a female patient were extracted and stored in Digital Imaging and Communications in Medicine (DICOM) format. Subsequently, the file was imported into the imaging processing software Materialise Mimics Version 20.0 (Materialise NV, Leuven, Belgium) for 3D design and modeling. First, the region of interest (meaning the unaffected ear) was manually selected with the paint brush tool, and the threshold was set to the soft-tissue range of −700 to +225 Hounsfield Units (HU). Repairing and smoothing tools were used during postprocessing for final refinement and artefact removal. The final 3D image (.stl) of the ear was exported into AutoCAD Software for image mirroring to the affected side on a skull model. A Boolean operation (Union-Subtract-Intersect) was performed on the customized auricular constructs to design uniform and systematic pores into the implant with AutoCAD. Based on the results of the limit test, a pore size of 0.67 mm was selected. Finally, the .stl files of the porous and non-porous implants were processed to G-code and exported to the Ultimaker 2+ 3D printer for additive “layer-by-layer” manufacturing as described above. Five porous and five solid auricular constructs were produced and evaluated for all three filament materials. The workflow of the design- and manufacturing process of the auricular constructs is illustrated in Figure 1.

### 4.8. Evaluation of the Macro- and Microdimensions of the Auricular Constructs

Five macro-dimensions of the final solid and porous auricular constructs were measured with the electronic calipers three times for each dimension, and descriptive statistics were calculated for each material. The PEL was measured from the highest point of the helix to the lowest point of the inferior border of the ear lobule. PEW describes the distance from the superaurale (the highest point of the upper edge of the helix of the ear) to the subaurale (the lowest point of the inferior border of the ear lobule). MEL was defined as the length from the Darwinian tubercle to the deepest point of the tragion. MEW was measured as the distance from the otobasion superius to the otobasion inferius, the upper and lower points at which the pinna is attached to the scalp, respectively. The height of the ear was measured at the mid-level of the tragus. Additionally, the two micro-dimensions (pore size and WRU) were evaluated for the porous auricular constructs under the light microscope (see above). The measurements of the dimensions in the AutoCAD software were regarded as reference.

### 4.9. Statistical Analysis

Statistical analysis was performed with the software GraphPad Prism 8 for Mac OS (GraphPad Software, San Diego, CA, USA). Depending on the Gaussian distribution (evaluated with a Shapiro–Wilk-test), either a Student’s *t*-test or a Mann–Whitney-U-test were used for statistical analysis. A one-way analysis of variance (ANOVA) was used to compare the dimensions of the three filament materials (ABS, PLA and PCL). Results are presented as the mean ± standard deviation (SD), and a *p*-value of <0.05 was considered as statistically significant.

## Figures and Tables

**Table 1:** Comparison between surgical procedures using different materials for ear reconstruction and the financial remunerations. The identical remunerations for different procedures in the G-DRG 2021 system does not display developmentary and material costs of medical implants. In-house production could offer a solution for this financial disbalance, while reducing the surgical complexity of the procedure.

Diagnosis (Code)	Microtia (Q17.2)		
Procedure	Ear reconstruction with autologous cartilage	Ear reconstruction with a self-made porous PCL implant	Ear reconstruction with a commercially available alloplastic implant
Surgical complexity of the procedure	High	Medium	Medium
CAD-CAM-assisted patient-customization	No	Yes	No
Donor site morbidity	Yes	No	No
G-DRG Procedural Code	5–187.2	5–187.4	5–187.4
Revenue (€) *	5695.2	3645.3	3645.3
Material costs/ implant (€)	0	0.56	1161.44 (incl. VAT) **
Equipment costs (€)	0	2309	0
Difference (€)	5695.2	1335.7	2483.87
Difference without equipment costs (€)	5695.2	3644.74	2483.87

* The overall revenue was calculated based on a 70 kg, 40-year-old female patient with an average hospital stay of 5 days, using the web calculator of the DRG-Research group (https://www.drg-research-group.de). ** The price calculation was based on the German list prices for the Ear Base Extended (CAT#8330) and Helical Rim (CAT#8328) MEDPOR two-piece implant by the Stryker Corporation (Kalamazoo, MI, USA).

**Table 2:** Descriptive statistics for the macro- and micro-dimensional accurary of the porous model scaffolds (*n* = 9; the significance threshold was set as *p* < 0.05, *p*-value for all statistical test were ns = not significant).

Material	ABS		PLA		PCL		ANOVA
Dimension (mm)	Mean	SD	Mean	SD	Mean	SD	
Edge Length	10.03	0.05	10.00	0.06	10.01	0.05	ns
Height	2.670	0.02	2.67	0.02	2.67	0.03	ns
WRU	0.661	0.01	0.66	0.01	0.67	0.01	ns
Pore Size	0.669	0.01	0.66	0.01	0.67	0.01	ns

**Table 3:** Statistics for measurements for the dimensions of the porous auricular constructs in mm (*n* = 5; the significance threshold was set as *p* < 0.05, *p*-value for all statistical test were ns = not significant).

Material	ABS			PLA			PCL			ANOVA
Dimension (mm)	Mean	SD	*t*-test	Mean	SD	*t*-test	Mean	SD	*t*-test	
PEL	51.74	0.12	0.97	51.75	0.11	0.84	51.73	0.15	0.91	ns
MEL	25.46	0.10	0.86	25.48	0.11	0.61	25.43	0.09	0.64	ns
PEW	28.39	0.13	0.89	28.37	0.11	0.84	28.39	0.12	0.89	ns
MEW	31.41	0.11	0.51	31.39	0.12	0.89	31.36	0.12	0.75	ns
Ear height	12.51	0.12	0.72	12.49	0.09	0.93	12.5	0.08	0.32	ns

**Table 4:** Descriptive statistics for measurements for the dimensions of the solid auricular constructs in mm (*n* = 5; the significance threshold was set as *p* < 0.05, *p*-value for all statistical test were ns = not significant).

Material	ABS			PLA			PCL			ANOVA
Dimension (mm)	Mean	SD	*t*-test	Mean	SD	*t*-test	Mean	SD	*t*-test	
PEL	51.73	0.10	0.84	51.75	0.11	0.88	51.72	0.15	0.77	ns
MEL	25.47	0.11	0.71	25.47	0.11	0.76	25.43	0.08	0.62	ns
PEW	28.41	0.12	0.80	28.37	0.13	0.58	28.38	0.11	0.70	ns
MEW	31.42	0.10	0.68	31.42	0.11	0.70	31.36	0.14	0.55	ns
Ear height	12.51	0.09	0.69	12.5	0.08	0.40	12.56	0.13	0.64	ns

**Table 5:** Overview of the rheological features potential application of the thermoplastic materials.

Filament Material	ABS	PLA	PCL
Company	Formfutura, Nijmegen, The Netherlands	Formfutura, Nijmegen, The Netherlands	3D4MAKERS, Haarlem, The Netherlands
Color	Blue	Black	White
Diameter	2.85 mm (+/− 0.05 mm)	2.85 mm (+/− 0.05 mm)	2.85 mm (+/− 0.05 mm)
Roundness	99%	99%	99%
Density	1.05 g/cm^3^	1.24 g/cm^3^	1.1 g/cm^3^
Properties	Strong and durable	Easy to print	Safe, nontoxic, and biodegradable
Application	End-use parts and casings	Prototypes	Medical implantation
Printing temp.	230–250 °C	180–210 °C	80–160 °C
Strength	High	Medium	Low
Flexibility	Low	Medium	High
Ease of printing	Medium	High	Low

## Data Availability

The data that support the findings of this study are available from the corresponding author upon reasonable request.
